# WFDC21P promotes triple-negative breast cancer proliferation and migration through WFDC21P/miR-628/SMAD3 axis

**DOI:** 10.3389/fonc.2022.1032850

**Published:** 2022-10-31

**Authors:** Yu-Bo Wei, Dong-Min Liang, Mei-Ling Zhang, You-Jie Li, Hong-Fang Sun, Qin Wang, Yan Liang, Yan-Mei Li, Ran-Ran Wang, Zhen-Lin Yang, Pingyu Wang, Shu-Yang Xie

**Affiliations:** ^1^ Key Laboratory of Tumor Molecular Biology, Department of Biochemistry and Molecular Biology, Binzhou Medical University, Yantai, Shandong, China; ^2^ Department of Physiology and Pathophysiology, School of Basic Medicine, Qingdao University, Qingdao, Shandong, China; ^3^ Department of Immune Rheumatism, Yantaishan Hospital, Yantai, Shandong, China; ^4^ Institute of Rehabilitation Medicine, School of Rehabilitation Medicine, Binzhou Medical University, Yantai, Shandong, China; ^5^ Department of Breast and Thyroid Surgery, Yantai Affiliated Hospital of Binzhou Medical University, Yantai, Shandong, China; ^6^ Department of Epidemiology, Binzhou Medical University, Yantai, Shandong, China

**Keywords:** WFDC21P, microRNA, N6-methyladenosine, epigenetics, triple-negative breast cancer

## Abstract

Long non-coding RNAs (lncRNAs) modulate cell proliferation, cycle, and apoptosis. However, the role of lncRNA-WFDC21P in the tumorigenesis of triple-negative breast cancer (TNBC) remains unclear. Results of this study demonstrated that WFDC21P levels significantly increased in TNBC, which was associated with the poor survival of patients. WFDC21P overexpression significantly promoted TNBC cell proliferation and metastasis. WFDC21P interacted with miR-628-5p, which further suppressed cell proliferation and metastasis by negatively regulating Smad3-related gene expression. Recovery of miR-628-5p weakened the roles of WFDC21P in promoting the growth and metastasis of TNBC cells. Moreover,N6-methyladenosine (m6A) modification upregulated WFDC21P expression in the TNBC cells. WFDC21P and its m6A levels were increased after methyltransferase like 3 (METTL3) overexpression but reduced after METTL3 silencing. The proliferation and metastasis of TNBC cells were promoted by METTL3 overexpression but suppressed by METTL3 silencing. This study demonstrated the vital roles of WFDC21P and its m6A in regulating the proliferation and metastasis of TNBC cells *via* the WFDC21P/miR-628/SMAD3 axis.

## Introduction

Breast cancer (BC) has the highest incidence rate and is the second cause of death in women worldwide (1). Triple-negative BC (TNBC), which features a lack of ER, PR, and HER2 expression, is the most aggressive subtype and accounts for 15%-20% of all BCs (2). Cytotoxic chemotherapy is the backbone of therapy against TNBC, and effective targeted therapies are currently available (3). Thus, the therapeutic targets of TNBC, such as noncoding RNA, need to be explored urgently.

Traditionally, epigenetic regulation regulates the noncoding RNA (ncRNA) function, DNA methylation, and histone regulation of chromatin (4). Epigenomic alterations can induce various tumor-promoting properties, including cell proliferation, metastasis, and apoptosis (5). N6-methyladenosine (m6A), the most common and abundant modification of mRNA, affects the efficiency of mRNA processing or transport (6), which is modulated by “writers,” “erasers,” and “readers” in eukaryotic cells. The “writer” METTL3/METTL14 heterodimer adds m6A, whereas the “eraser” ALKBH5 removes from thousands of transcripts in a single-stranded context (7, 8). However, the underlying mechanisms of m6A in TNBC remain unknown.

ncRNA, including microRNA (miRNA), long non-coding RNA (lncRNA), and circular RNA modulate cell proliferation, cell cycle, apoptosis, and DNA methylation (9). lncRNAs, as transcripts longer than 200 nucleotides, function as master regulators for gene expression and participate in various disease processes, including carcinogenesis (10). They are key oncogenes or tumor-suppressor genes that affect cancer occurrence and development through lncRNA–miRNA or lncRNA–mRNA interaction. Various lncRNAs are reportedly dysregulated in TNBC (11–13). MIR503HG expression was reduced in TNBC and its overexpression inhibited the cell migration ability and proliferation through regulating the miR-224-5p/HOXA9 axis (11). lncRNA HUMT recruits Y-box binding protein 1 and enhances the expression of vascular endothelial growth factor C in TNBC (12). NAMPT-AS is an oncogenic lncRNA that epigenetically activates NAMPT to promote the proliferation of TNBC cells (13).

lncRNA WFDC21P plays pivotal roles in rheumatoid arthritis, multiple sclerosis, and gastric cancer (14–16), but its roles in TNBC development are unclear. A recent study has reported that WFDC21P is upregulated by miR-4293 and plays an oncogenic role in lung cancer (17). In the present study, we aimed to investigate the roles of WFDC21P and its m6A modification in TNBC tumorigenesis, and demonstrated that m6A modification increased WFDC21P levels, which was regulated by the “writer” METTL3. WFDC21P functioned as the sponge of miR-628 to upregulate Smad3 expression. This study indicated that WFDC21P promoted TNBC cell proliferation *in vitro* and *in vitro via* the WFDC21P/miR-628/SMAD3 axis.

## Materials and methods

### Clinical samples

A total of 26 TNBC patients who underwent a mastectomy were recruited to the study at the Affiliated Yantaishan Hospital and the Yantai affiliated Hospital of Binzhou Medical University (Yantai, China). All specimens were pathologically confirmed as TNBC and did not receive radiotherapy or chemotherapy prior to surgery, the demographic characteristics of cases were shown in **Supplemental Table 1**. After resection, the tumor and adjacent tissues were frozen by liquid nitrogen, and the specimens were immediately stored at −80°C. This study was approved by the Medical Ethics Committee of Binzhou Medical University. The study procedures were fully explained to patients before study inclusion, and written informed consents were acquired from all enrolled patients.

### Bioinformatics analysis

WFDC21P levels were determined in multiple cancers using Gene Expression Profiling Interactive Analysis (GEPIA) websites (http://gepia.cancer-pku.cn/). The Kaplan–Meier curve was used to test the association of WFDC21P with overall survival (OS). The binding sites between WFDC21P and miR-628 or between Smad3-3’UTR and miR-628 were analyzed by using the miRDB (http://mirdb.org/miRDB) or TargetScan 7.1 database (http://www.targetscan.org).

### Cell culture

BT549 and MCF-10A cells were purchased from Shanghai Institute of Cell Biology (Shanghai, China). SUM159 cells were obtained from American Type Culture Collection. These cells were cultured in Dulbecco’s modified Eagle’s medium supplemented with 10% fetal bovine serum (FBS). All cells were cultured in a humidified atmosphere containing 5% CO_2_ at 37°C.

### Construction of lentiviral vectors

WFDC21P-overexpression and siRNA-WFDC21P lentiviral vectors were constructed and produced as previously described (18). H1 promoter and shRNA sequences were cloned into a blunt-ended *Pac*I-digested FUGW plasmid (kindly provided by Dr. Zack Wang, Massachusetts General Hospital, Harvard University). *EcoRI*–*EcoRV* element containing WFDC21P was cloned into the E*coR*I–E*coR*V site of the FUGW plasmid to construct the overexpression vector. 293T cells were used for lentivirus production. The lentivirus was harvested, filtered, and added to the recipient cells in accordance with standard protocols.

### Cell transfection and infection

TNBC cells (1×10^5^ cells/well) were cultured in 6-well plates with 2 mL culture medium for 16h. For transfection, miR-628 inhibitor (ASO-628, 5′-CCUCUAGUAAAUAUGUCAGCAU-3′) and control oligos were transfected into TNBC cells using Lipofectamine 2000 (Invitrogen, Carlsbad, CA, USA) following the manufacturer’s instructions. For infection, TNBC cells (1×10^5^ cells/well) were treated with 1×10^5^ FU lentivirus which overexpressed WFDC21P or miR-628-5p (5′-AUGCUGACAUAUUUACUAGAGG-3′).

### *In situ* hybridization

The experiment was performed with an RNA FISH kit (No. F11201, GenePharma, Shanghai, China) in accordance with the manufacturer’s instructions. The 5′-FAM-labeled probe for WFDC21P was designed and synthesized by GenePharma (Shanghai, China). In a typical procedure, the cells were fixed with 4% paraformaldehyde for 20 min, permeabilized, incubated with a 1 μM probe, and then hybridized at 37°C overnight. The nuclei were stained with DAPI for 5 min, and the cells were observed under a confocal microscope (LEICA TCS SPE, Leica, Dresden, Germany).

### Quantitative real-time PCR (qRT-PCR)

Total RNA (or small RNA) from TNBC tissues or cells was extracted by RNAiso Plus (or RNAiso for small RNA, Takara, Dalian, China) as previously described (17). Then, RNA (< 1 μg) was reverse transcribed into cDNA with a SPARKscriptIIRT Plus Kit (AG0304-B, SpakeJade, Jinan, China). The primers for detection are shown in **Supplemental Table 2**. GAPDH or 5S rRNA was used as an endogenous control to normalize the expression of target genes. Each sample was analyzed in triplicate. qRT-PCR was performed using a Quantitect SYBRGreen Kit (204243, QIAGEN, Germany) on a StepOnePlus Real-time system (ThermoFisher, MD, USA).

### Hoechst staining

TNBC cells were seeded in 24-well plates. Hoechst 33342 dye (160 μL, Solarbio, Beijing, China) was added to each well at 48 h after transfection, and then incubated for 20 min. The fluorescence of each well was detected by an EVOS M700 Cell Imaging System (Thermo Fisher Scientific, USA) to count the number of alive cells.

### Transwell assay

Cells in 100 µL of medium with 10% (FBS) were reseeded into the upper chambers of 24-well Transwell plates (Corning, NY, USA), and the lower chamber was filled with 600 µL of 30% FBS medium as previously described (17).

### Immunoblotting

Immunoblotting was performed as described previously (17–19). Protein was extracted from SUM159 cells using 1× RIPA buffer (P0013B, Beyotime, Shanghai, China). Subsequently, the proteins were separated by 10% sodium dodecyl sulfate–polyacrylamide gel electrophoresis and then electro-transferred onto PVDF membranes, which were incubated with primary antibodies overnight at 4°C. The protein bands were detected using enhanced chemiluminescence substrate (P0018M, BeyoECL Plus, Beyotime, China). The antibodies used were as follows: mouse anti-human E-cadherin (1:500, BF0219, Affinit); rabbit anti-human SNAI1 (1:500, 13099-1-AP, Proteintech); rabbit anti-human SMAD3 (1:500, BM3919, BOSTER); rabbit anti-human p-Smad3 (1:500, AP0328, Bioworld); rabbit anti-human N-Cadherin (1:500, No.48495-2), rabbit anti-human METTL3 (1:500, No.31591), and rabbit anti-human GAPDH (1:3000, MB001) all from Bioworld Technology, Ltd).

### RNA immunoprecipitation (RIP) and m6A methylated RIP (MeRIP)

RIP and MeRIP assays were performed using a Magna RIP RNA-Binding Protein Immunoprecipitation Kit (17-700, Millipore, Billerica, MA, USA) in accordance with the manufacturer’s instructions. Antibodies for RIP assays of AGO2 and m6A were obtained from CST (2897 and 56593, respectively, Boston, MA, USA).The coprecipitated RNAs were detected using qRT-PCR.

### Luciferase assay

Luciferase levels were analyzed using the Luciferase Assay System (E1500, Promega, WI, USA). In a typical procedure, cells were transfected with luciferase-expressed vectors or controls. After 48 h, the cells were lysed with 200 μL of lysis buffer and incubated at room temperature for 15 min. Then, 96-well plates were added with 70 μL of lysate and 20μL of Luciferase Assay Reagent II. Luciferase activity was measured with a chemiluminescence instrument (Infinite 200 PRE, Tecan Austria GmbH).

### TNBC cell xenografts

All animal experiments were approved by the Committee on the Ethics of Animal Experiments of Binzhou Medical University and performed in accordance with the Guidelines for the Care and Use of Laboratory Animals of National Institutes of Health guidelines. Animals were grouped by simple randomization using a random number table. SUM159 cells were treated with lentivirus to stably express shRNA or WFDC21P. A total of 1 × 10^7^ cells were injected subcutaneously into the backs of BALB/C-nude mice (aged 6–8 weeks, HFK Bio-Technology, Beijing, China). The primary tumors were measured daily with a caliper. One month later, the mice were euthanized by intraperitoneally injecting a barbiturate.

### Statistics

Statistical significance of data was analyzed using SPSS 22.0 software (IBM Corp., Armonk, NY, USA). Normally distributed data are presented as the mean ± SD of at least three independent experiments, and Student’s t-test and ANOVA were used for the mean comparison of two and multiple groups, respectively. Abnormally distributed data are expressed as the median (interquartile range), and Mann–Whitney U and Kruskal–Wallis H tests were used to compare two and multiple groups, respectively. Differences with *p* < 0.05 were considered statistically significant.

## Results

### WFDC21P was overexpressed in BC and TNBC tissues

**Figure 1:**
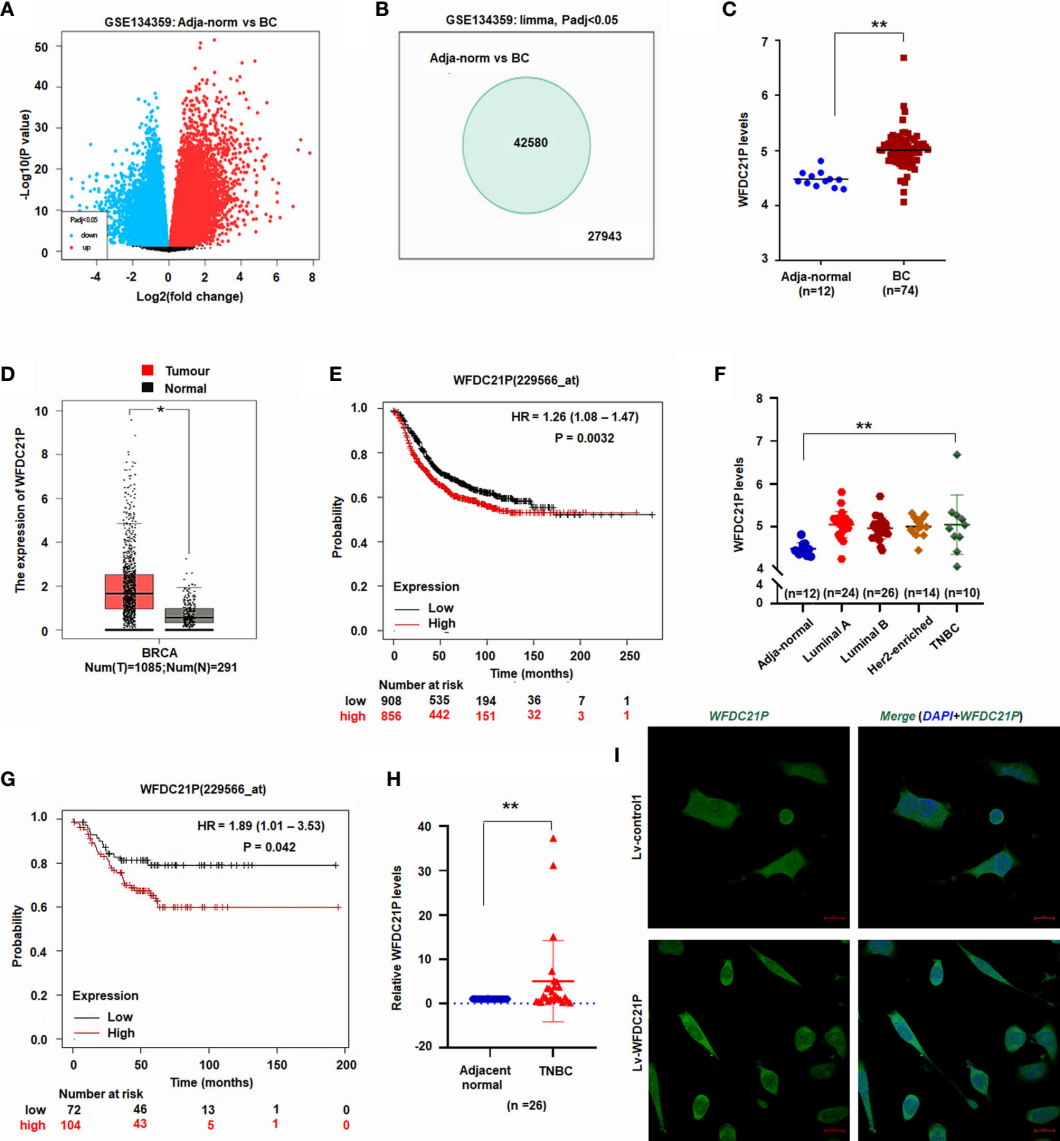
Expression of WFDC21P in BC. **(A)** Volcano plot of lncRNA microarray datasets GSE134359. **(B)** Number of different lncRNA gene expression. **(C)** WFDC21P expression between BC and adjacent normal tissues from GSE134359. Data are shown as median (interquartile range), **p < 0.01, Mann–Whitney U test. **(D)** WFDC21P levels were higher in BC tissues. BC tissues, n=1085; normal tissues, n=291; *p < 0.05. **(E)** Kaplan–Meier plotter analysis of the overall survival of patients with BC. **(F)** WFDC21P levels were higher in TNBC tissues than in normal adjacent tissues from GSE134359. Normal, n = 12; Luminal A, n = 24; Luminal B, n = 26; Her2-enriched, n = 14; TNBC tissues, n = 10; Kruskal–Wallis H tests, **p < 0.01. **(G)** Kaplan–Meier Plotter analysis of the overall survival of patients with TNBC. **(H)** WFDC21P expression was estimated by qRT-PCR in TNBC tissues (n = 26) and adjacent normal tissues. Data are shown as median (interquartile range), **p < 0.01; n=26, Mann–Whitney U test. **(I)** *In situ* hybridization detection of WFDC21P. Green color in cell indicates the expression of WFDC21P. bar = 20 μm.

It is reported that WFDC21P plays an oncogenic role in non-small cell lung cancer (17). Here, the roles of WFDC21P in BC and TNBC were investigated. First, lncRNA microarray datasets from gene expression analyses were downloaded from the NCBI Gene Expression Omnibus through accession code GSE134359 (https://www.ncbi.nlm.nih.gov/gds/?term=GSE134359). The expression of 42,580 lncRNAs, including WFDC21P, was significantly higher in BC tissues (n = 74) than in adjacent normal tissues (n = 12) (**Figures 1A–C**).

GEPIA is a web-based tool that delivers fast and customizable functionalities based on TCGA and GTEx data (20). To further investigate its levels in BC tissues, WFDC21P expression in BC tissues was further analyzed using GEPIA (http://gepia.cancer-pku.cn/). The data from GEPIA showed that the WFDC21P levels were significantly higher in BC tissues (n=1085) than in normal control tissues (n=291, *p* < 0.05; **Figure 1D**). The Kaplan–Meier plotter was used to assess the effect of different genes on survival in cancers. We then analyzed the association of WFDC21P expression with the OS of patients with BC through the Kaplan–Meier plotter website (http://www.kmplot.com/analysis/). Results indicated that high WFDC21P expression in patients with BC corresponded with poor survival compared with low levels (p = 0.0032; **Figure 1E**).

Next, we studied the roles of WFDC21P in TNBC, which is a particularly aggressive subtype of BC and has high invasiveness and metastatic potential (21). Based on the data of GSE134359, the expression of WFDC21P was significantly higher in TNBC tissues (n=10) than in adjacent normal tissues (n=12) (**Figure 1F**). The Kaplan–Meier plotter further confirmed that high WFDC21P expression in patients with TNBC had a poor survival (*p* = 0.042; **Figure 1G**). qRT-PCR results showed that WFDC21P expression was significantly higher in TNBC tissues than in control tissues (*p* < 0.01; **Figure 1H**). These results indicated that WFDC21P might play important roles in the tumorigenesis of TNBC.

### WFDC21P promoted the proliferation and metastasis of TNBC cells through Smad3-related genes

**Figure 2:**
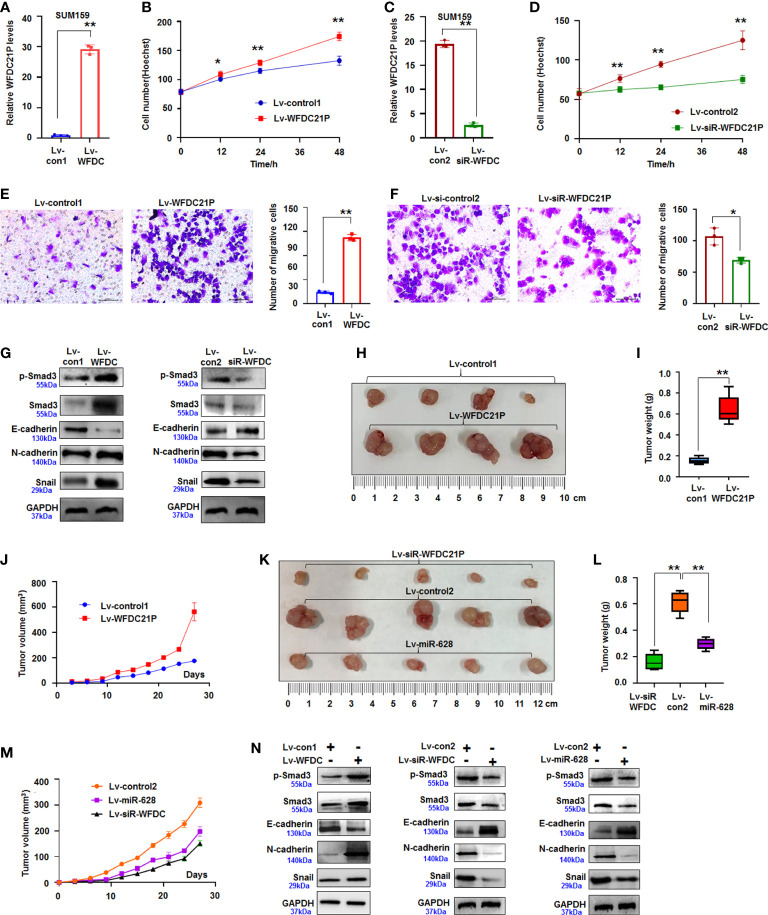
WFDC21P promoted SUM159 cell proliferation and metastasis. **(A)** qRT-PCR analysis of WFDC21P expression in lv-WFDC21P-overexpressed cells. Data are expressed as the mean ± SD of triplicate experiments. **p < 0.01; Student’s t-test. **(B)** Hoechst staining assay illustrated that WFDC21P promoted SUM159 cell proliferation. Data are expressed as the mean ± SD of triplicate experiments. *p < 0.05, **p < 0.01; ANOVA. **(C)** qRT-PCR analysis of WFDC21P expression in the lv-siR-WFDC21P-treated cells. Data are expressed as the mean ± SD of triplicate experiments. **p < 0.01; Student’s t-test. **(D)** Hoechst staining assay. siRNA-WFDC21P decreased SUM159 cell proliferation. Data were expressed as mean ± SD for triplicate experiments. **p < 0.01; ANOVA. **(E, F)** Metastasis of SUM159 cells infected with lv-WFDC21P or lv-siR-WFDC21P or with controls. Data are presented as the mean ± SD of triplicate experiments. *p < 0.05, **p < 0.01; Student’s t-test. **(G)** immunoblotting detection of cell metastasis-related proteins. **(H–J)** Tumor size, weight, and volume changes were measured after nude mice were subcutaneously injected with SUM159 cells stably expressed with WFDC21P or control. Data are presented as the mean ± SD or median (interquartile range). n=4, **p < 0.01; Student’s t-test or Mann–Whitney U test. **(K–M)** Tumor size, weight, and volume changes were measured after nude mice were subcutaneously injected with cells stably expressed with siRNA-WFDC21P, or miR-628, or control. Data are presented as the mean ± SD or median (interquartile range). n=4 **p < 0.01; ANOVA or Kruskal-Wallis H test. **(N)** immunoblotting detection of cell metastasis-related proteins in xenografts.

We first explored WFDC21P expression in TNBC cells (SUM159 and BT549) and control cells (MCF-10A) to further determine the roles of WFDC21P in the proliferation and metastasis of TNBC cells. Results showed that WFDC21P expression was higher in TNBC cells than in MCF-10A cells (**Supplementary Figure S1**). After SUM159 cells were infected with lv-WFDC21P, *in situ* hybridization and qRT-PCR detection showed that WFDC21P was overexpressed in TNBC cells (**Figures 1I**, **2A**), and cell proliferation was significantly promoted compared with lv-control-treated cultures, as estimated by Hoechst staining assay (**Figure 2B**). When WFDC21P expression was suppressed by siRNA, cell proliferation was obviously inhibited in the lv-siR-WFDC21P-treated cultures compared with the lv-control-treated cells (**Figures 2C, D**). Transwell migration assay showed that SUM159 cell metastasis was significantly enhanced by WFDC21P overexpression (**Figure 2E**) but inhibited by WFDC21P suppression (**Figure 2F**) compared with the control. Experiments in BT549 also confirmed that cell metastasis was promoted by WFDC21P overexpression (**Supplementary Figures 2A–C**), but suppressed by WFDC21P downregulation (**Supplementary Figures 2D–F**) compared with control treatment. Experiments in control MCF-10A cells demonstrated that the effect of WFDC21P overexpression and siRNA-WFDC21P on the growth of MCF-10A cells was not changed significantly compared with control treatment (**Supplementary Figures 2G, H**).

Smad3 regulates the metastasis of tumor cells and the expression of epithelial–mesenchymal–transition genes (22). In the present study, the expression of Smad3-related E-cadherin, N-cadherin, and Snail was detected by immunoblotting. Results showed that the expression of p-Smad3, Smad3, N-cadherin, and Snail increased but that of E-cadherin decreased in the WFDC21P-overexpressed SUM159 cells compared with the control-treated cells. Knockdown of WFDC21P expression by siRNA downregulated the expression of p-Smad3, Smad3, N-cadherin, and Snail were reduced but upregulated the expression of E-cadherin was in SUM159 cells compared with the control (**Figure 2G**). This finding indicated that WFDC21P regulated the expression of Smad3-related genes during TNBC cell metastasis.

Furthermore, SUM159 cells stably expressing WFDC21P or siRNA-WFDC21P were subcutaneously injected into the backs of BALB/C-nu nude mice to produce xenografts and study the roles of WFDC21P in TNBC tumorigenesis *in vivo*. After 4 weeks, the volumes of transplanted TNBC tumors stably expressing WFDC21P considerably increased, and the weights obviously increased than those in the control groups (**Figures 2H, I**; **Supplementary Figure 3A**). The tumor-growth curve confirmed that WFDC21P overexpression promoted TNBC proliferation *in vivo* (**Figure 2J**). However, siRNA-WFDC21P treatment decreased the volumes and weights of TNBC xenografts (**Figures 2K, L**; **Supplementary Figure 3B**). It also demonstrated that WFDC21P downregulation inhibited TNBC growth *in vivo* (**Figure 2M**). Immunoblotting detection demonstrated that p-Smad3, Smad3, N-cadherin, and Snail increased but E-cadherin decreased in the WFDC21P-overexpressed xenografts. The expression of p-Smad3, Smad3, N-cadherin, and Snail decreased, whereas that of E-cadherin increased in the siRNA-WFDC21P-treated xenografts compared with the controls (**Figure 2N**). The above results suggested that WFDC21P can enhance the cell proliferation and metastasis of TNBC cells.

### WFDC21P regulated miR-628-5p expression

**Figure 3:**
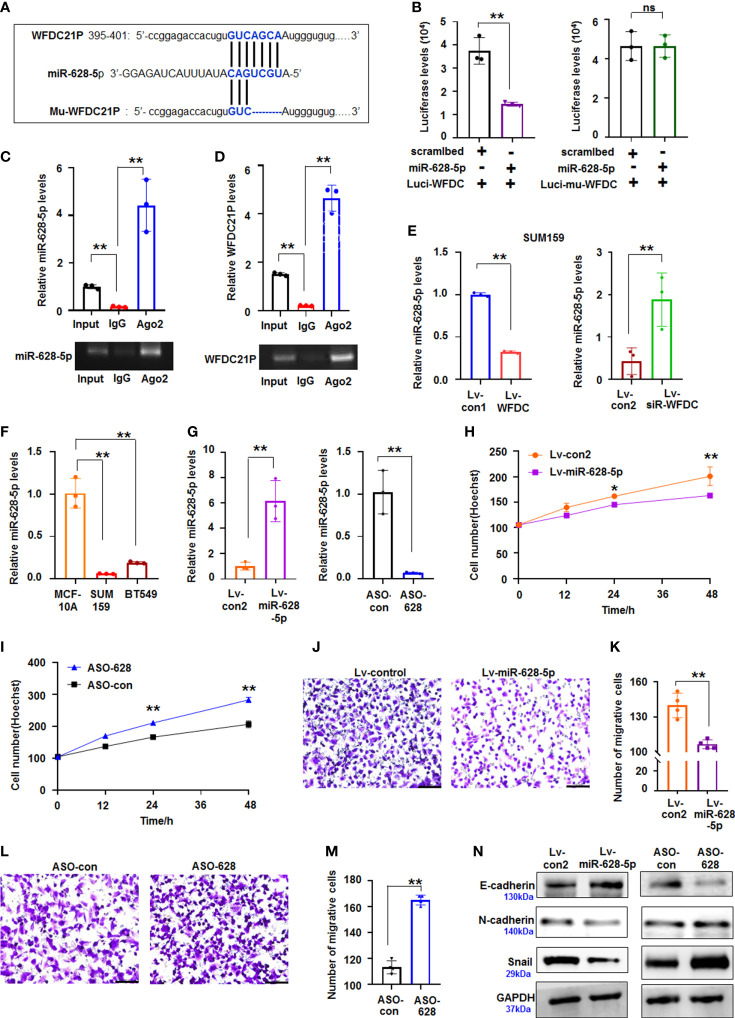
miR-628-5p was regulated by WFDC21P and inhibited TNBC proliferation and metastasis. **(A)** The site of WFDC21P or Mu-WFDC21P mRNA matched with miR-628-5p. **(B)** SUM159 cells were treated with pc3.1-luci-WFDC21P (or mu-luci-WFDC21P) and miR-628-5p or controls. Data are expressed as the mean ± SD of triplicate experiments. **p < 0.01, Student’s t-test. ns means “no significance". **(C, D)** RNA immunoprecipitation assays. Levels of miR-628-5p and WFDC21P were estimated using qRT-PCR, respectively. Data are expressed as the mean ± SD of triplicate experiments. **p < 0.01, ANOVA. **(E)** WFDC21P overexpression or siRNA-WFDC21P affected miR-628-5p levels in SUM159 cells, respectively. Data are expressed as the mean ± SD of triplicate experiments. **p < 0.01, Student’s t-test. **(F)** miR-628-5p levels were analzyed by qRT-PCR in SUM159, BT549, and MCF-10A cells. Data were expressed as mean ± SD for triplicate experiments. **p < 0.01, ANOVA. **(G)** miR-628-5p expression was analyzed after SUM159 cells were treated with lv-miR-628-5p -expression virus or ASO-628 oligos. Data are expressed as the mean ± SD of triplicate experiments. **p < 0.01, Student’s t-test. **(H, I)** Hoechst staining assay illustrated that miR-628-5p suppressed or ASO-628 promoted SUM159 cell proliferation. Data are expressed as the mean ± SD of triplicate experiments. *p < 0.05, **p < 0.01; ANOVA. **(J, K)** miR-628-5p overexpression inhibited SUM159 cell metastasis. Data are expressed as the mean ± SD of triplicate experiments. **p < 0.01; Student’s t-test. **(L, M)** ASO-628 promoted SUM159 cell metastasis. Data are expressed as mean ± SD of triplicate experiments. **p < 0.01; Student’s t-test. **(N)** immunoblotting detection.

Growing evidence supports that lncRNAs play vital roles in cancer progression by competitively binding to matched miRNAs, thereby regulating their cognate genes (23). However, limited data are available regarding the interactions between lncRNAs and miRNAs related to the growth and metastasis of TNBC cells. The roles of WFDC21P-related miRNAs in TNBC were further explored using miRDB bioinformatics prediction. Results showed that WFDC21P can potentially bind to miR-628-5p (**Figure 3A**). Luciferase assay was performed in SUM159 cells cotransfected with pc3.1-luci-WFDC21P and miR-628-5p. The luciferase levels decreased in the pc3.1-luci-WFDC21P+miR-628-5p-treated cells compared with the control, but no obvious luciferase changes were found in the mutant vector (Mu-luci-WFDC21P)+miR-628-5p-treated cells (**Figure 3B**). RNA immunoprecipitation was performed to study the interaction between WFDC21P and miR-628-5p. Results showed that WFDC21P and miR-628-5p were pulled down by Ago2 antibody (**Figures 3C, D**), indicating that WFDC21P interacted with miR-628-5p by binding to Ago2. Moreover, miR-628-5p expression was decreased by WFDC21P overexpression but increased by WFDC21P knockdown in SUM159 and BT549 cells (**Figure 3E**; **Supplementary Figure 3C**). These results suggested that WFDC21P can interact with miR-628-5p and negatively regulate its levels.

### miR-628-5p suppressed the proliferation and metastasis of TNBC cells

Aberrant miRNA expression has been reported in TNBC, exhibiting oncogenic and tumor-suppressive miRNAs (24). In the present study, we further investigated the roles of miR-628-5p in regulating the proliferation and metastasis of TNBC cells. Compared with the high levels of WFDC21P in TNBC cells, miR-628-5p levels were decreased in SUM159 and BT549 cells (**Figure 3F**). miR-628-5p overexpression significantly suppressed SUM159 cell proliferation compared with the control, whereas ASO-628 (miR-628-5p inhibitor) promoted cell proliferation compared with that in the control-treated cultures (**Figures 3G–I**). The number of migrative cells was lower in the lv-miR-628-5p-overexpressing cultures compared with the lv-con-treated SUM159 cells (**Figures 3J, K**). Inhibiting miR-628-5p expression by ASO-628 can promote SUM159 cell metastasis compared with the control (**Figures 3L, M**). The migrative experiments of BT549 cells also confirmed that miR-628-5p can suppress the metastasis of TNBC cells (**Supplementary Figures 4A–D**). Furthermore, cell metastasis-related proteins (N-cadherin, E-cadherin, and Snail) were analyzed to investigate the detailed mechanism by which miR-628-5p inhibits the metastasis of TNBC cells. The expression of E-cadherin increased but those of N-cadherin and Snail decreased in the lv-miR-628-5p-overexpressing cells.E-cadherin expression decreased but N-cadherin and Snail expression increased in the ASO-628-5p-treated cells (**Figure 3N**).

Subsequently, SUM159 cells stably expressed miR-628-5p and controls were used to produce xenografts to study the role of miR-628-5p in TNBC *in vivo*. The volumes and weights were lower in the miR-628-5p-treated TNBC xenografts than in the controls (**Figures 2K-M**, **Supplementary Figure 3D**). Immunoblotting demonstrated that the expression of p-Smad3, Smad3, N-cadherin, and Snail decreased but that of E-cadherin increased in the miR-628-5p-treated xenografts (**Figure 2N**). The above results suggested that miR-628-5p suppressed the proliferation of TNBC cells.

### miR-628-5p negatively regulated Smad3 expression

**Figure 4:**
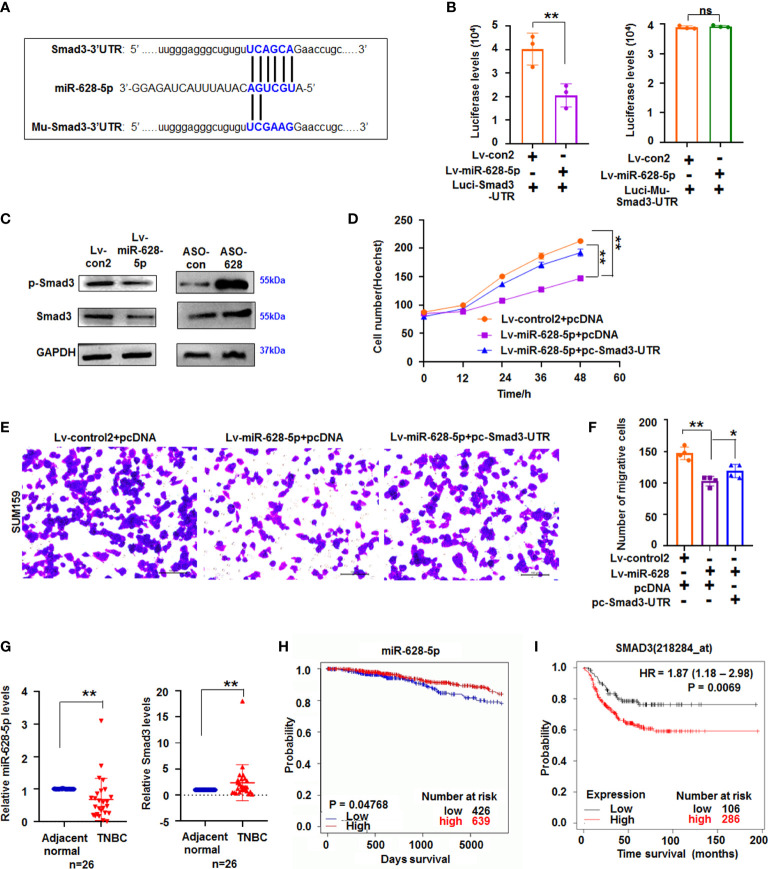
miR-628-5p regulated Smad3 expression. **(A)** The site of Smad3-3’UTR or Mu-Smad3-3’UTR targeted by miR-628-5p. **(B)** Luciferase levels were analyzed in pc3.1-luci-Smad3-3ʹ UTR and miR-628-5p-treated cells. Data are expressed as the mean ± SD of triplicate experiments. **p < 0.01, Student’s t-test. ns means “no significance”. **(C)** immunoblotting detection. **(D)** Effect Smad3-3′UTR treatment on the role of miR-628-5p in SUM159 cell growth using Hoechst staining assay. Data are expressed as the mean ± SD of triplicate experiments. *p < 0.05, **p < 0.01; ANOVA. **(E, F)** Effect of Smad3-3′UTR treatment on the role of miR-628-5p in regulating SUM159 cell metastasis. Data are expressed as the mean ± SD of triplicate experiments. **p < 0.01; ANOVA. **(G)** Expression of miR-628-5p and Smad3 in TNBC tissues (n=26) was analyzed by qRT-PCR. Data are shown as median (interquartile range), **p < 0.01; n=26, Mann–Whitney U test. **(H, I)** Kaplan–Meier plotter analysis of the relationship between levels of miR-628-5p (or Smad3) with the overall survival of patients with BC, respectively.

miRNA can suppress mRNA translation or expression by binding the complementary bases in the 3ʹ untranslated region (3ʹUTR) of its target gene (25). TargetScanHuman prediction online (http://www.targetscan.org/vert_71/) showed that miR-628-5p can potentially bind the sequences of Smad3-3ʹUTR (**Figure 4A**). Next, luciferase reporter assay showed that luciferase activity decreased in the cells co-transfected with Luci-Smad3-3’UTR and miR-628-5p, but miR-628-5p did not reduce the luciferase levels in the Luci-Mu-Smad3-3′UTR (with mutant sites of 3′UTR)-treated cultures (**Figure 4B**). These results indicated that miR-628-5p can regulate luciferase expression through the 3′UTR of Smad3. Moreover, immunoblotting results demonstrated that Smad3 protein expression was suppressed by miR-628-5p (**Figure 4C**), confirming that miR-628-5p negatively regulated Smad3 expression. To further test the role of miR-628-5p in regulating Smad3, we determined whether the recovery of Smad3-3′UTR can rescue the inhibitory effect of miR-628-5p on TNBC cell growth. Results showed that the recovery of Smad3-3′UTR can attenuate the suppressing role of miR-628-5p in SUM159 cell growth (**Figure 4D**). Smad3-3′UTR treatment also restored the SUM159 and BT549 cell metastasis reduced by miR-628-5p treatment (**Figures 4E, F**; **Supplementary Figure 5**). These results confirmed that Smad3 was a target of miR-628-5p.

To further study their roles in TNBC tumorigenesis, the expression of miR-628-5p and Smad3 in TNBC tissues was measured using qRT-PCR. Results showed that the expression of miR-628-5p significantly decreased (*p* < 0.01, n = 26), whereas that of Smad3 significantly increased (*p* < 0.01, n=26) in TNBC tissues compared with adjacent normal tissues (**Figure 4G**). We then analyzed the effect of their expression on survival of patients with breast invasive carcinoma by the using the Kaplan–Meier plotter. Results indicated that low miR-628-5p expression in patients with breast invasive carcinoma corresponded to poor survival compared with high expression (*p* = 0.04768, **Figure 4H**, http://www.oncomir.org/). The data also confirmed that high Smad3 expression in patients with TNBC corresponded to poor survival (*p* = 0.0069, **Figure 4I**).

### WFDC21P/miR-628/Smad3 axis modulated the tumorigenesis of TNBC

The abovementioned results also demonstrated that WFDC21P overexpression can reduce miR-628-5p but increase p-Smad3 and Smad3 expression. Moreover, miR-628-5p could negatively regulate Smad3 expression. These results indicated the role of the WFDC21P/miR-628-5p/Smad3 axis in TNBC tumorigenesis.

**Figure 5:**
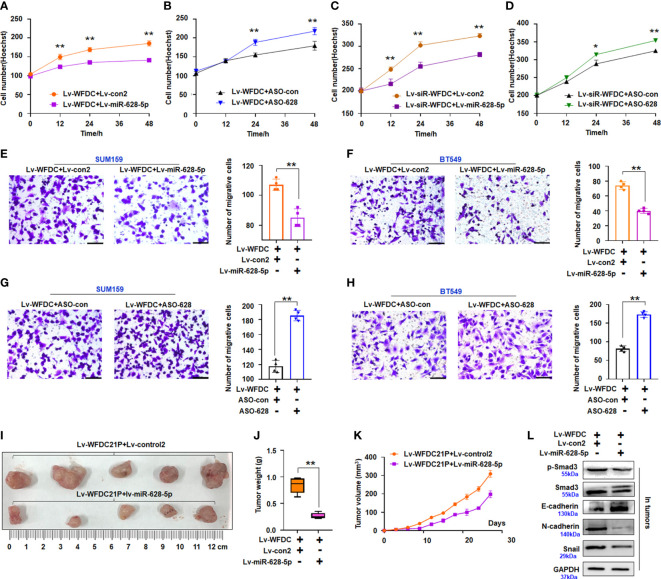
Effect of miR-628-5p on oncogenic roles of WFDC21p. **(A–D)** Hoechst staining assay. miR-628-5p treatment attenuated but ASO-628 treatment enhanced the promoting effect of WFDC21P on SUM159 cell proliferation. miR-628-5p treatment increased but ASO-628 treatment weakened the suppressive effect of siRNA-WFDC21P on SUM159 cell proliferation. Data are expressed as the mean ± SD of triplicate experiments. *p < 0.05, **p< 0.01; Student’s t-test. **(E–H)** Effect of miR-628-5p treatment on the promoting effect of WFDC21P on TNBC cell metastasis. Data are expressed as the mean ± SD of triplicate experiments. **p < 0.01; Student’s t-test. **(I–K)** Tumor size, weight, and volume changes were measured after xenografts were treated with miR-628-5p+WFDC21P. Data are expressed as mean ± SD or median (interquartile range). **p < 0.01; Student’s t-test or Mann–Whitney U test. **(L)** Immunoblotting detection of cell metastasis-related proteins in xenografts.

To further study the role of this axis, we determined whether or not miR-628-5p recovery can reduce the role of WFDC21P in promoting the growth or metastasis of TNBC cells. miR-628-5p treatment attenuated the role of WFDC21P in promoting SUM159 cell proliferation compared with the lv-control treatment (**Figure 5A**). When miR-628-5p expression was inhibited by ASO-628 (inhibitor), the role of WFDC21P in promoting SUM159 cell proliferation was strengthened (**Figure 5B**). miR-628-5p treatment increased, whereas ASO-628 treatment attenuated the suppressive role of siRNA-WFDC21P in SUM159 cell proliferation compared with the lv-control treatment (**Figures 5C, D**). These results indicated that miR-628-5p recovery reduced the role of WFDC21P in promoting TNBC cell proliferation. miR-628-5p treatment also attenuated the role of WFDC21P in promoting SUM159 and BT549 cell metastasis compared with the lv-control treatment (**Figures 5E, F**), whereas ASO-628 strengthened the role of WFDC21P in promoting SUM159 and BT549 cell proliferation compared with the lv-control treatment (**Figures 5G, H**). Moreover, the suppressive role of siRNA-WFDC21P in SUM159 or BT549 cell metastasis was enhanced by miR-628-5p treatment but weakened by ASO-628 treatment (**Supplementary Figures 6A-D**), indicating that miR-628-5p recovery weakened the role of WFDC21P in TNBC cell metastasis.

Furthermore, we determined whether or not miR-628-5p treatment can reduce the role of WFDC21P in cell growth *in vivo*. The volumes and weights of the lv-miR-628-5p+WFDC21P-treated xenografts decreased compared with the control treatment (**Figures 5I, J**). The tumor growth curve demonstrated that miR-628-5p treatment could reduce the role of WFDC21P *in vivo* (**Figure 5K**). Moreover, the expression of p-Smad3, Smad3, N-cadherin, and Snail was downregulated while that of E-cadherin was upregulated in the Lv-miR-628-5p+WFDC21P-treatment xenografts (**Figure 5L**).

Together, the results indicated that the WFDC21P/miR-628/SMAD3 axis modulated TNBC tumorigenesis, and miR-628-5p recovery reduced the oncogenic role of WFDC21P in TNBC cell growth.

### m6A modification was associated with WFDC21P upregulation in TNBC cells

**Figure 6:**
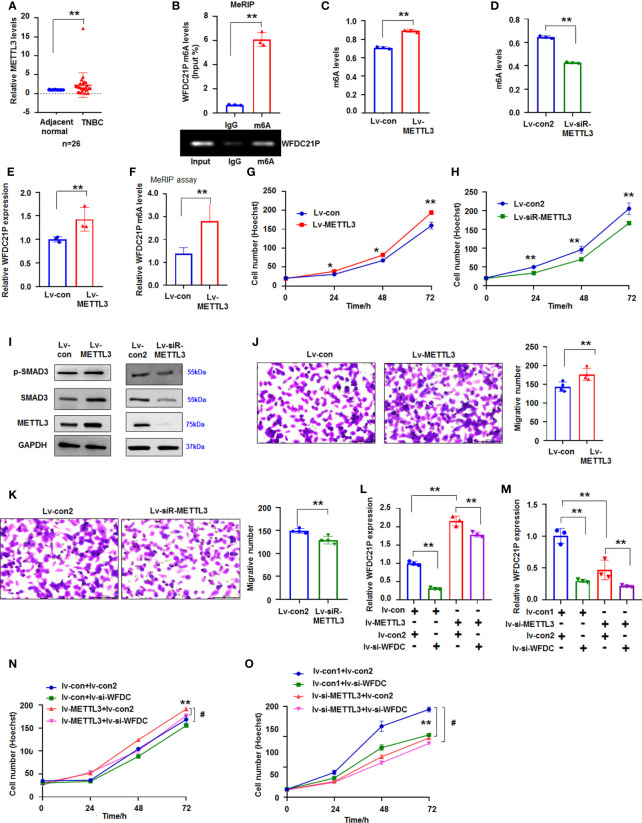
Effect of METTL3 on WFDC21p m6A and cell growth. **(A)** qRT-PCR detection of METTL3 levels in TNBC tissues. Data are shown as median (interquartile range), **p < 0.01; n=26, Mann–Whitney U test. **(B)** MeRIP analysis of WFDC21p m6A levels in SUM159 cells. Data are expressed as the mean ± SD of triplicate experiments. **p < 0.01; Student’s t-test. **(C, D)** Effect of METTL3 overexpression or downregulation on m6A levels in SUM159 cells, respectively. Data are expressed as the mean ± SD of triplicate experiments. **p < 0.01; Student’s t-test. **(E)** The effect of METTL3 overexpression on WFDC21P levels in SUM159 cells. Data are expressed as the mean ± SD of triplicate experiments. **p < 0.01; Student’s t-test. **(F)** MeRIP assay. METTL3 overexpression increased WFDC21P m6A levels in SUM159 cells. Data are expressed as the mean ± SD of triplicate experiments. **p < 0.01; Student’s t-test. **(G, H)** Effect of METTL3 overexpression or downregulation on SUM159 cell proliferation, respectively. Data are expressed as the mean ± SD of triplicate experiments. *p < 0.05, **p < 0.01; Student’s t-test. **(I)** immunoblotting analysis. **(J, K)** Effect of METTL3 overexpression or downregulation on SUM159 cell metastasis, respectively. Data are expressed as the mean ± SD of triplicate experiments. **p < 0.01; Student’s t-test. **(L, M)** qRT-PCR analysis of WFDC21P levels. Data are expressed as the mean ± SD of triplicate experiments. **p < 0.01; ANOVA. **(N, O)** siRNA-WFDC21P reduced the effect of METTL3 on cell growth. Data were expressed as mean ± SD. **p < 0.01, ^#^ *p* < 0.001; ANOVA.

METTL3, a key factor of the large N6-adenosine-methyltransferase complex, is crucial to m6A modification in lncRNA (26). qRT-PCR results indicated that METTL3 expression was significantly higher (*p* < 0.01, n=26) in TNBC tissues than in adjacent normal tissues (**Figure 6A**). Then, we determined whether m6A was associated with the upregulation of WFDC21P in TNBC cells. Results from the online bioinformatics database m6Avar demonstrated three WFDC21P m6A sequence motifs in the exon region (at ch17: 60085172, 60085180, and 60086035) (http://m6avar.renlab.org). MeRIP further showed that WFDC21P was modified by m6A (**Figure 6B**). METTL3 overexpression increased m6A levels (**Figure 6C**; **Supplementary Figure 7A**), but METTL3 downregulation decreased m6A levels in SUM 159 cells (**Figure 6D**; **Supplementary Figure 7B**). METTL3 overexpression increased WFDC21P expression (**Figure 6E**). MeRIP assay also further supported that METTL3 overexpression could increase WFDC21P m6A levels (**Figure 6F**).

Subsequently, we studied the effect of METTL3 on cell proliferation. Hoechst staining showed that METTL3 overexpression promoted SUM159 cell growth, but siRNA-METTL3 suppressed SUM159 cell proliferation (**Figures 6G, H**). Immunoblotting results demonstrated that METTL3 overexpression increased but siRNA-METTL3 suppressed p-Smad3 and Smad3 levels in SUM159 cells (**Figure 6I**), indicating that METTL3 influenced cell proliferation possibly by regulating p-Smad3 and Smad3.

METTL3 upregulation also promoted cell metastasis, whereas siRNA-METTL3 inhibited SUM159 cell metastasis (**Figures 6J, K**). These results demonstrated that WFDC21P and m6A levels were affected by METTL3. We then determined whether blocking WFDC21P could weaken the role of METTL3 in cell proliferation. Results showed that siRNA-WFDC21P reduced the effect of METTL3 on upregulating WFDC21P expression, which further suppressed the role of METTL3 in promoting SUM159 cell proliferation (**Figures 6L–O**).

## Discussion

**Figure 7:**
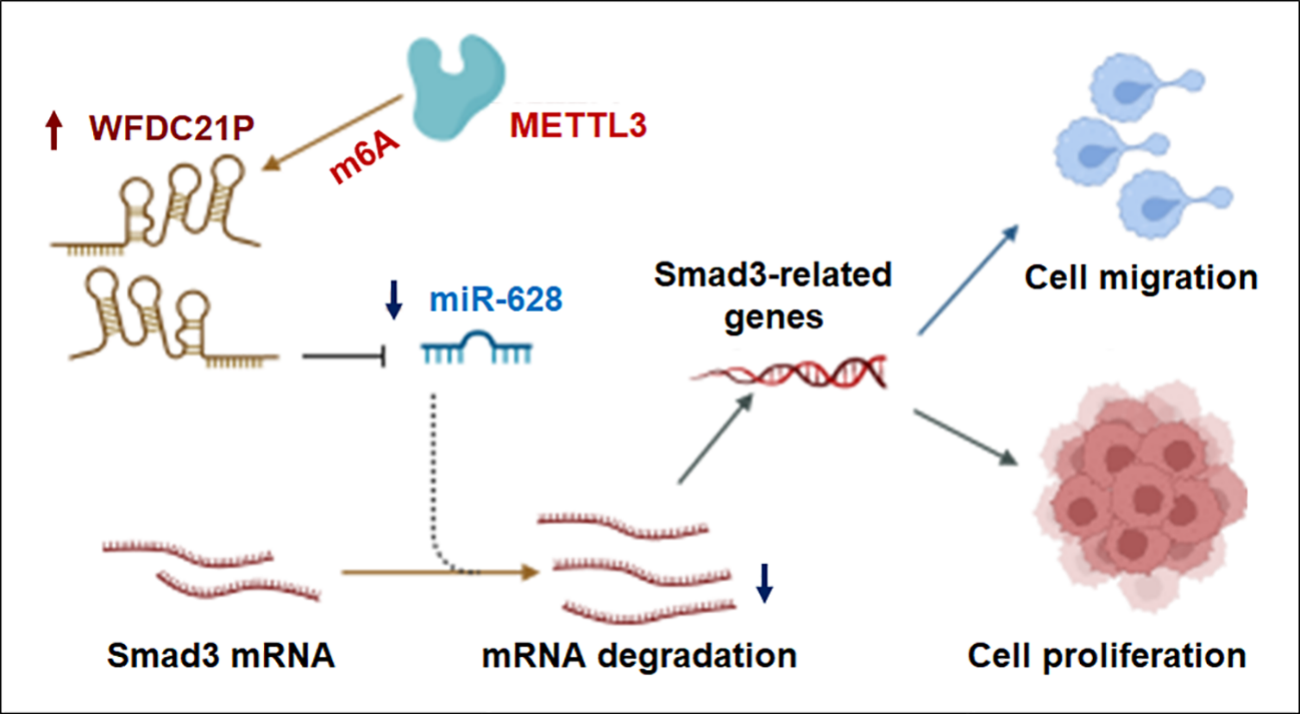
Proposed model by which METTL3 promoted lncRNA-WFDC21P levels and triple-negative breast cancer progression. Through the WFDC21P/miR-628-5p/Smad3 axis, WFDC21P significantly promoted BC cell proliferation and metastasis and negatively regulating miR-628-5p, whereas miR-628-5p suppressed BC cell proliferation and metastasis by negatively regulating Smad3-related gene expression. Moreover, METTL3 overexpression increased WFDC21P and its m6A levels and further regulated BC cell proliferation and metastasis.

lncRNAs play crucial roles in the epigenetic regulation of different types of cancer cells (27). As the most abundant epigenetic modification on lncRNAs, m6A has been linked to diverse effects on lncRNA function in multiple biological and pathological processes, especially in cancer (28). In the present study, we investigated the effects of WFDC21P and m6A modification on TNBC progression, and found that WFDC21P expression was significantly upregulated in TNBC tissues. Moreover, high WFDC21P expression in patients with TNBC corresponded to poor survival compared with its low expression. Through the WFDC21P/miR-628-5p/Smad3 axis, WFDC21P significantly promoted cell proliferation and metastasis by negatively regulating miR-628-5p, whereas miR-628-5p suppressed TNBC cell proliferation and metastasis by negatively regulating Smad3-related gene expression. Furthermore, m6A was associated with the upregulation of WFDC21P in TNBC cells. METTL3 overexpression increased WFDC21P expression and its m6A levels, which further promoted TNBC cell proliferation and metastasis (**Figure 7**).

Although molecular-targeting therapies against tumors have achieved remarkable results, an effective therapy for TNBC is lacking (29). Hence, novel therapeutic targets are urgently needed to improve TNBC treatment. Recently, lncRNAs have become a hot spot in ncRNA research, and increased evidence supports the role of lncRNAs in TNBC progression. For example, lncRNA GAS5 in TNBC patients is related to tumor resistance to PTX, which induces apoptosis by regulating the miR-378a-5p/SUFU signal pathway (30). GATA3-AS1 increases in TNBC tissues and cells and contributes to immune evasion through the miR-676-3p/COPS5 axis (31). lncRNA-PCAT6 upregulates VEGFR2 expression *via* ceRNA and participates in the angiogenesis of TNBC *via* the VEGFR/AKT/mTOR signaling pathway (32). Similarly, we found that WFDC21P was upregulated in TNBC, which is related to poor clinical prognosis. Upregulated WFDC21P can promote TNBC cell proliferation *in vitro* and *in vivo via* the WFDC21P/miR-628/SMAD3 axis.

Increasing evidence further supports that lncRNAs act as competing endogenous RNAs in the progression of cancer progression through the lncRNA-miRNA-mRNA network (33). lncRNAs could interact with miRNA, which is related to tumorigenesis in various types of cancer including TNBC (34–36). lncRNA-HEIH functions as a ceRNA to target SOCS1 *via* the direct sponging of miR-4458 and regulates TNBC cell proliferation (34). lncRNA AFAP1-AS1 could regulate cell proliferation, migration and invasion of TNBC cells by inhibited the miR-195/miR-545 axis (35). WEE2-AS1 was up-regulated in TNBC cells and demonstrated an oncogenic function through WEE2-AS1/miR-32-5p/TOB1 axis (36). In present study, our results further demonstrated that lncRNA WFDC21P facilitated the proliferation and migration of TNBC cells through the WFDC21P/miR-628-5p/Smad3 axis.

miRNAs, approximately 19-25 nucleotides in length, regulate the expression of their targeted genes at the post-transcriptional level. The dysregulation of miRNAs is involved in multiple biological processes, including the proliferation, migration, differentiation, and apoptosis of cancer cells (37). miR-628-5p functions as a tumor-suppressive gene in pancreatic ductal adenocarcinoma by regulating AKT, in gastric cancer by targeting PIN1, and in cervical cancer by regulating JAG1 (38–40). However, the roles of miR-628-5p in TNBC remain unclear. In present study, bioinformatics analysis showed that WFDC21P had a potential binding site to miR-628-5p. qRT-PCR showed that WFDC21P overexpression downregulated miR-628-5p expression in TNBC cells. Moreover, miR-628-5p overexpression inhibited TNBC cell proliferation by negatively regulating Smad3-related factors *in vitro* and *in vivo*.

Smad3 plays important roles in the cell proliferation, invasion and metastasis of cancer cells, such as lung cancer (41), colorectal cancer (42), and chordoma (43). TGF-β could promote the progression of tumorigenesis in BC and facilitate cancer cell migration and invasion through the Smad3 signaling pathway (44). In the present study, Smad3 was found to be a novel target of miR-628-5p. The downregulation of Smad3 and EMT-related proteins by miR-628-5p suppressed TNBC cell migration, and WFDC21P regulated TNBC cell migration through the WFDC21P/miR-628-5p/Smad3 axis. Furthermore, Smad3 regulates the expression of epithelial–mesenchymal–transition (EMT) genes, which is associated with cancer metastasis (22). The common features of EMT are that E-cadherin was downregulated, while N-cadherin were overexpressed. Similarly, our results supported that overexpression of WFDC21P could decrease E-cadherin levels but increase N-cadherin and Snail levels through miR-628-5p/Smad3 axis.

m6A modification has become an epigenetics research hotspot in recent years because of its crucial roles in the expression, function, and stabilization of lncRNA transcripts in human cancers (45). Mettl3 and Mettl14 are core m6A writers catalyzing the methylation of adenosines and regulating the functions of lncRNAs (46). METTL14 could regulate the m6A level of XIST, and the loss of METTL14 is related to poor prognosis of patients with colorectal cancer (47). METTL14 overexpression could suppress the proliferation and migration of papillary thyroid cancer cells by regulating the lncRNA OIP5-AS1 and MEK/ERK pathways (48). METTL3 upregulates MALAT1 expression by affecting its m6A modification, and MALAT1 promotes BC cell migration and invasion *via* the MALAT1/miR-26b/HMGA2 axis (49). METTL3 could also upregulate the expression of LINC00958 by promoting its RNA transcript stability, affecting miR-378a-3p to promote YY1 in BC tumorigenesis (50). In the present study, METTL3 was upregulated in TNBC tissues, and its overexpression promoted TNBC cell proliferation through the upregulation of WFDC21P and m6A levels.

This study demonstrated the vital role of WFDC21P in regulating the cell proliferation and metastasis of TNBC *via* the WFDC21P/miR-628/SMAD3 network. WFDC21P downregulated the miR-628 expression and significantly contributed to TNBC development and tumorigenesis by regulating the Smad3 signal pathway. Moreover, METTL3 overexpression increased WFDC21P expression and promoted TNBC cell proliferation by regulating m6A modification. This study provided novel and potential effective therapeutic targets for TNBC treatment. Nevertheless, this study has several limitations. More clinical specimens should be included to investigate the function of WFDC21P in the future study. The details of WFDC21P-related signaling pathways are needed to be further explored for the tumorigenesis of TNBC.

## Data availability statement

The original contributions presented in the study are included in the article/**Supplementary Material**. Further inquiries can be directed to the corresponding authors.

## Ethics statement

The studies involving human participants were reviewed and approved by The Medical Ethics Committee of Binzhou Medical University. The patients/participants provided their written informed consent to participate in this study. The animal study was reviewed and approved by the Committee on the Ethics of Animal Experiments of Binzhou Medical University.

## Author contributions

S-YX conceived the study. PW and S-YX designed experiments. Y-BW, and D-ML constructed lentiviral vectors and performed FACS experiments. Y-BW, D-ML, M-LZ, and H-FS performed in site hybridization, qRT-PCR, immunoblotting experiments. YL performed bioinformatics analysis. QW, R-RW, and Y-JL produced mouse model. Y-ML and Z-LY collected clinical samples. Y-BW and D-ML performed the experiments of luciferase reporter assay, cell transfection, cell proliferation, and cell migration. PW analyzed the data. D-ML and Y-JL estimated RNA immunoprecipitation assay and m6A methylated RNA immunoprecipitation. PW analyzed the experiments with human samples. S-YX and PW prepared figures. Y-BW and S-YX wrote the manuscript. All authors contributed to the article and approved the submitted version.

## Funding

The present study was supported by the National Natural Science Foundation of China (No.81772281, 31371321), the Shandong Science and Technology Committee (No. ZR2019MH022, ZR2020KH015), the Education Department of Shandong Province (2019KJK014, 2021KJK005), and the Shandong Province Taishan Scholar Project (no. ts201712067).

## Acknowledgments

We thank professional editing support from ShineWrite.com (service@shinewrite.com) for editing the English text of a draft of this manuscript. We created the proposed model by the help of the Biorender.

## Conflict of interest

The authors declare that the research was conducted in the absence of any commercial or financial relationships that could be construed as a potential conflict of interest.

## Publisher’s note

All claims expressed in this article are solely those of the authors and do not necessarily represent those of their affiliated organizations, or those of the publisher, the editors and the reviewers. Any product that may be evaluated in this article, or claim that may be made by its manufacturer, is not guaranteed or endorsed by the publisher.
