# Supplementary material for: WFDC21P promotes triple-negative breast cancer proliferation and migration through WFDC21P/miR-628/SMAD3 axis
Source: Front Oncol. 2022 Oct 31;12:1032850. doi: 10.3389/fonc.2022.1032850 (PMC9659817; doi:10.3389/fonc.2022.1032850)
Supplement: Supplementary file 1 [file DataSheet_1.pdf]

WFDC21P promotes triple-negative breast cancer proliferation and migration through WFDC21P/miR-628/SMAD3 axis

Supplemental Figures and Table

Supplemental Figures 1-7 and figure legends

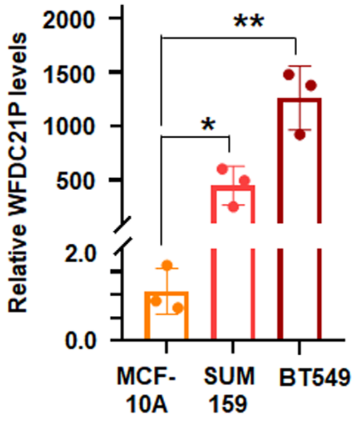

Figure S1 qRT-PCR analysis of WFDC21P expression in TNBC cells.

WFDC21P levels in SUM159 and BT549 was higher than that in MCF-10A control cells. Data represent the mean±SD; for triplicate experiments. *$p < 0.05$, ** $p < 0.01$; ANOVA.

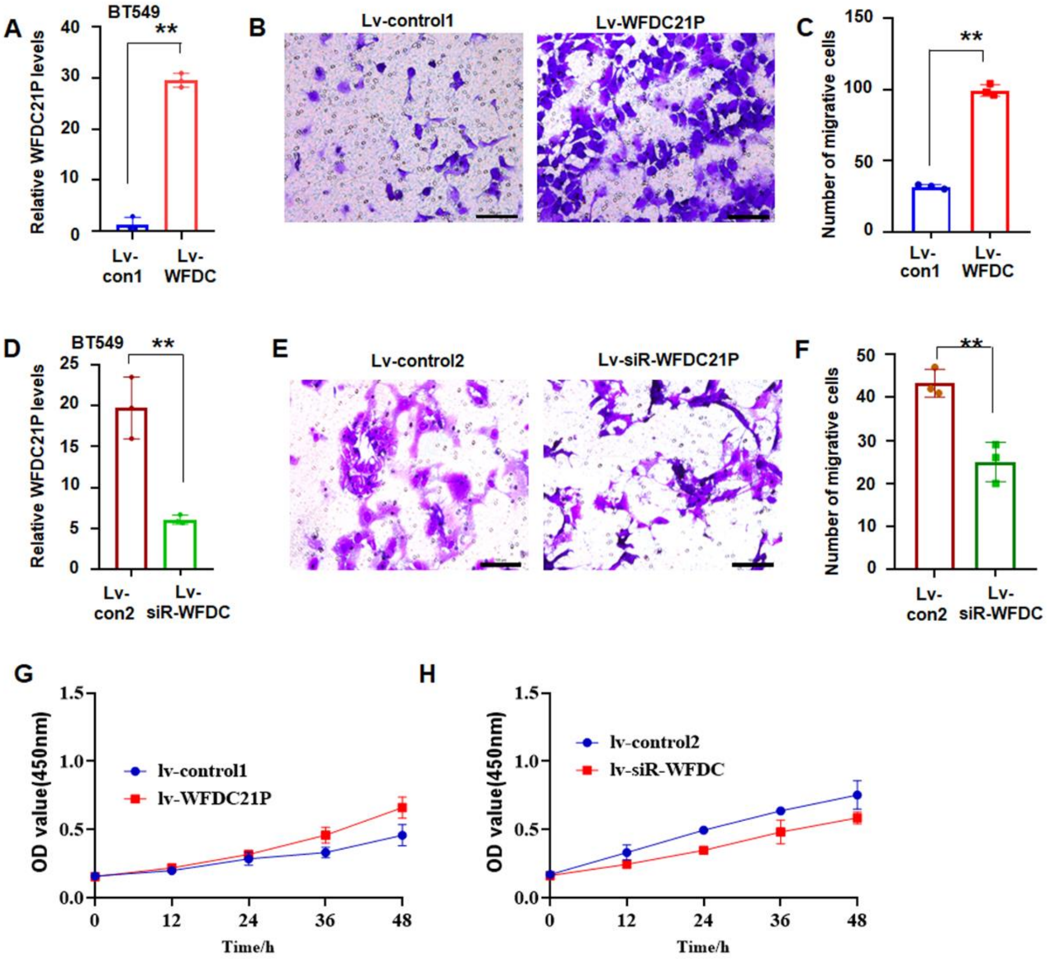

Figure S2 WFDC21P promoted to BT549 cell metastasis and its expression in xenografts. **(A)** qRT-PCR analysis of WFDC21P expression in lv-WFDC21P-overexpressed BT549 cells. Data were expressed as mean ± SD for triplicate experiments. **$p < 0.01$; Student's t-test. **(B,C)** WFDC21P overexpression promoted BT549 cell metastasis. Data were expressed as mean ± SD for triplicate experiments. * $p < 0.05$, ** $p < 0.01$; Student's t-test. **(D)** qRT-PCR analysis. WFDC21P expression was analyzed in lv-siR-WFDC21P-treated BT549 cells. Data were expressed as mean ± SD for triplicate experiments. **$p < 0.01$; Student's t-test. **(E,F)** WFDC21P-siRNA suppressed BT549 cell metastasis. Data were expressed as mean ± SD for triplicate experiments. * $p < 0.05$, ** $p < 0.01$; Student's t-test. **(G,H)** The effect of WFDC21P overexpression and siRNA-WFDC21P on the proliferation of MCF-10A cells.

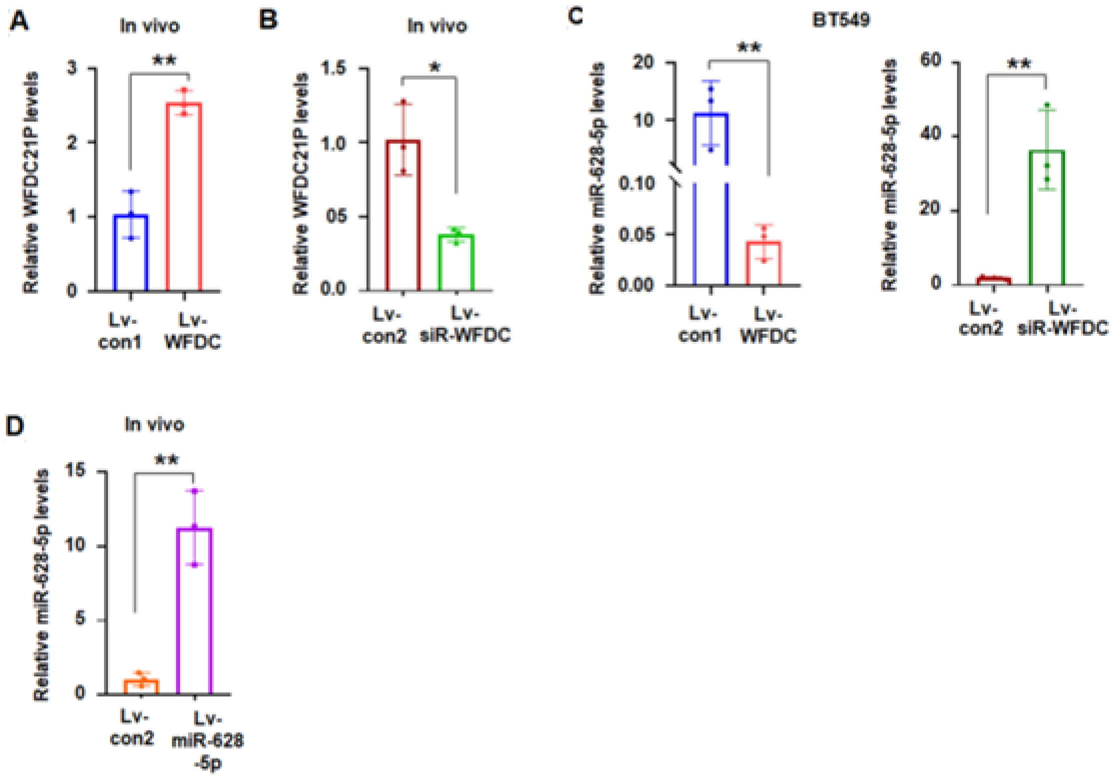

Figure S3 The expression of WFDC21P and miR-628-5p.

(A,B) WFDC21P expression was estimated in xenografts stably expressed WFDC21P or siRNA-WFDC21P. **(C)** WFDC21P overexpression or siRNA-WFDC21P affected miR-628-5p levels in BT549 cells. Data were expressed as mean ± SD. ** $p < 0.01$, Student's t-test. **(D)** miR-628-5p expression in xenografts stably expressed miR-628-5p. *$p < 0.05$, **$p < 0.01$; Student's t-test.

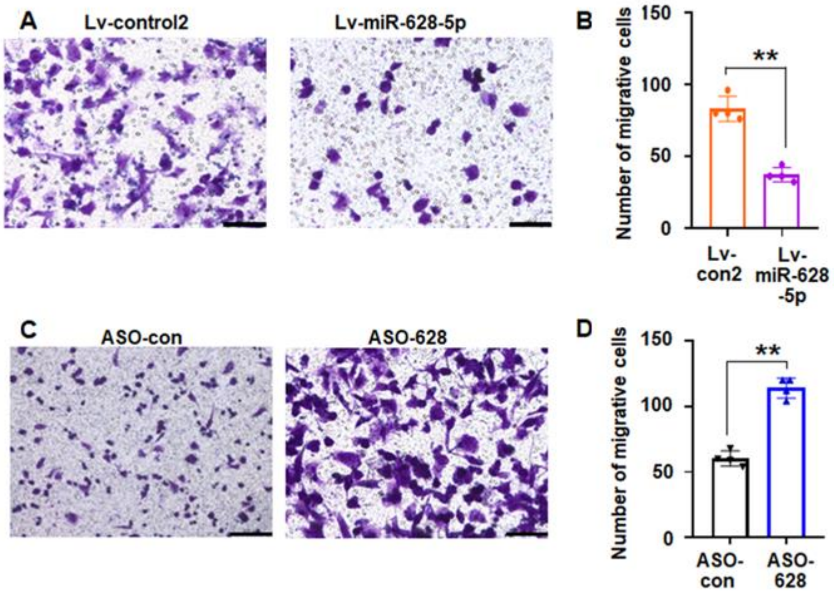

Figure S4 miR-628-5p inhibited BT549 cell metastasis.

(A,B) miR-628-5p overexpression inhibited BT549 cell metastasis. **(C,D)** ASO-628-5p promoted BT549 cell metastasis. Data were expressed as mean ± SD for triplicate experiments. ***p* < 0.01; Student's t-test.

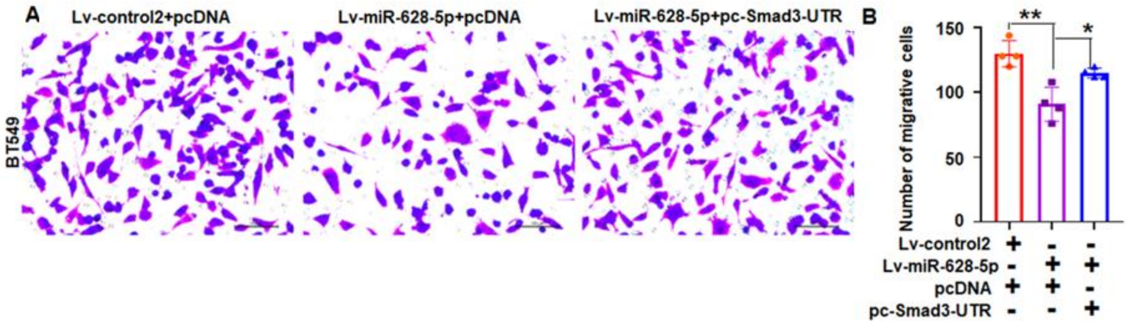

Figure S5 SUM159 cell metastasis detection.

(A) The effect of Smad3-3′UTR on the role of miR-628-5p in regulating BT549 cell metastasis. **(B)** Data were expressed as mean ± SD for triplicate experiments. ** $p < 0.01$; ANOVA.

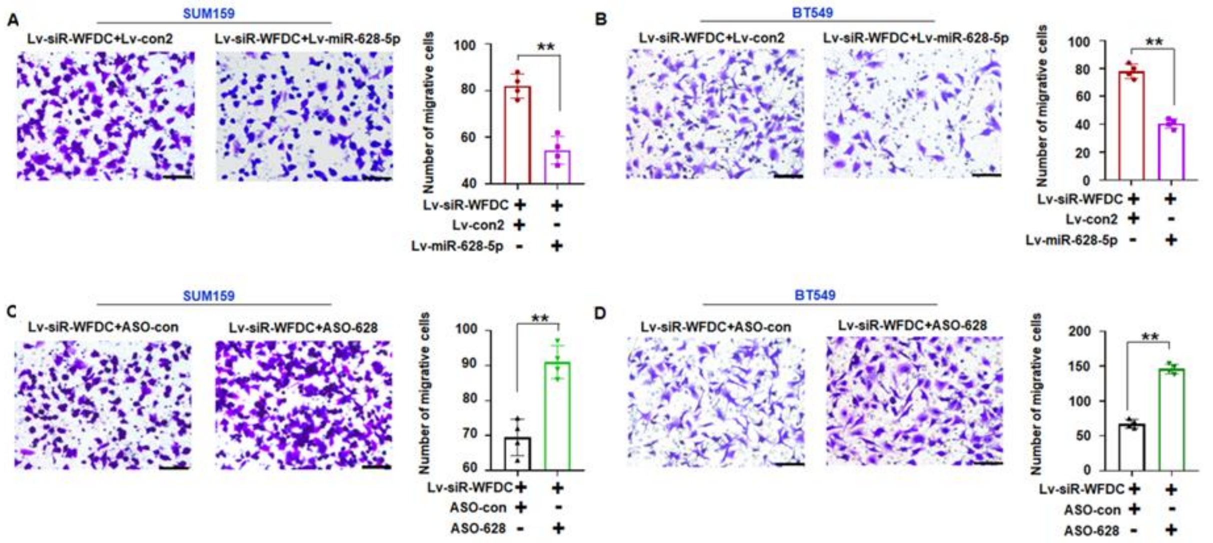

Figure S6 miR-628-5p treatment affected WFDC21P-promoting TNBC cell metastasis. **(A,B)** miR-628-5p treatment strengthened siRNA-WFDC21P-suppressing SUM159 and BT549 cell metastasis. **(C,D)** ASO-628 treatment attenuated siR-WFDC21P-inhibiting SUM159 and BT549 cell metastasis. Data were expressed as mean ± SD for triplicate experiments. ***p* < 0.01; Student's t-test.

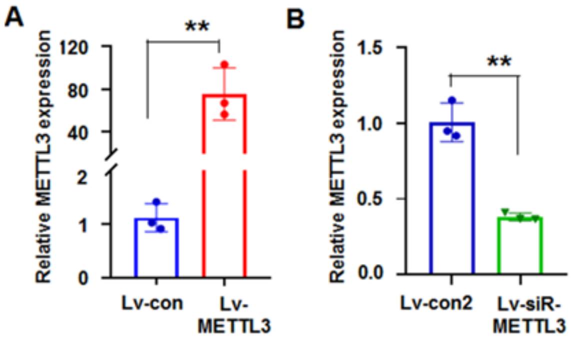

Figure S7 METTL3 expression was analyzed in SUM159 cells.

(A) METTL3 overexpression and **(B)** downregulation after siRNA-METTL3 treatment were detected in SUM159 cells, respectively. ** $p < 0.01$; Student's t-test.

Supplemental Tables

Supplemental Table 1. The demographic characteristics of cases

**Group**		**Case<br>n=26**
**Age(mean±SD)**		53.0±11.1
**height(mean±SD)**		159.4±6.3
**Smoking**		
	**Yes**	1 (3.8)
	**No**	25(96.2)
**Drinking**		
	**Yes**	3 (11.5)
	**No**	23 (88.5)
**TNM stage**		
	**Ⅰ-Ⅱ stage**	18 (69.2)
	**Ⅲ-Ⅳ stage**	8 (30.8)

Supplemental Table 2. The primers used in this study

Genes	Sequences (5’ to 3’)
WFDC21P- sense	CCAAGACCTGAGCCCTGTAA
antisense	ATAGAGGTGGCTGTCTGATGCT
GAPDH- sense	GTCTTCACCACCATGGAGAAGG
antisense	GCCTGCTTCACCACCTTCTTGA
MiR-628-5p	ATGCTGACATATTTACTAGAGG
5s rRNA	GCCATACCACCCTGAACG
RTQ	AACATGTACAGTCCATGGATG
